# Conserved B-Cell Epitopes among Human Bocavirus Species Indicate Potential Diagnostic Targets

**DOI:** 10.1371/journal.pone.0086960

**Published:** 2014-01-27

**Authors:** Zhuo Zhou, Xin Gao, Yaying Wang, Hongli Zhou, Chao Wu, Gláucia Paranhos-Baccalà, Guy Vernet, Li Guo, Jianwei Wang

**Affiliations:** 1 MOH Key Laboratory of Systems Biology of Pathogens and Christophe Mérieux Laboratory, IPB, CAMS-Fondation Mérieux, Institute of Pathogen Biology (IPB), Chinese Academy of Medical Sciences (CAMS) & Peking Union Medical College (PUMC), Beijing, People's Republic of China; 2 Fondation Mérieux, Lyon, France; Fondazione IRCCS Policlinico San Matteo, Italy

## Abstract

**Background:**

Human bocavirus species 1–4 (HBoV1–4) have been associated with respiratory and enteric infections in children. However, the immunological mechanisms in response to HBoV infections are not fully understood. Though previous studies have shown cross-reactivities between HBoV species, the epitopes responsible for this phenomenon remain unknown. In this study, we used genomic and immunologic approaches to identify the reactive epitopes conserved across multiple HBoV species and explored their potential as the basis of a novel diagnostic test for HBoVs.

**Methodology/Principal Findings:**

We generated HBoV1–3 VP2 gene fragment phage display libraries (GFPDLs) and used these libraries to analyze mouse antisera against VP2 protein of HBoV1, 2, and 3, and human sera positive for HBoVs. Using this approach, we mapped four epitope clusters of HBoVs and identified two immunodominant peptides–P1 (^1^MSDTDIQDQQPDTVDAPQNT^20^), and P2 (^162^EHAYPNASHPWDEDVMPDL^180^)–that are conserved among HBoV1–4. To confirm epitope immunogenicity, we immunized mice with the immunodominant P1 and P2 peptides identified in our screen and found that they elicited high titer antibodies in mice. These two antibodies could only recognize the VP2 of HBoV 1–4 in Western blot assays, rather than those of the two other parvoviruses human parvovirus B19 and human parvovirus 4 (PARV4). Based on our findings, we evaluated epitope-based peptide-IgM ELISAs as potential diagnostic tools for HBoVs IgM antibodies. We found that the P1+P2-IgM ELISA showed a higher sensitivity and specificity in HBoVs IgM detection than the assays using a single peptide.

**Conclusions/Significance:**

The identification of the conserved B-cell epitopes among human bocavirus species contributes to our understanding of immunological cross-reactivities of HBoVs, and provides important insights for the development of HBoV diagnostic tools.

## Introduction

Human bocavirus (HBoV) was first identified in nasopharyngeal samples of children with acute respiratory-tract infections (ARTIs) in 2005. This first virus was later designated as HBoV species 1 (HBoV1) [1]. HBoV1 is frequently detected in respiratory tract samples of children with upper or lower respiratory tract infections (URTIs/LRTIs) [2]–[5]. Three additional human bocavirus species, HBoV2, 3, and 4 were recently identified in fecal samples, but appear to be rare in respiratory tract samples and less prevalent in the population [6]–[10].

HBoV is frequently co-detected with other viruses and persists in the nasopharynx [11], [12]. Thus, the extent of correlation between HBoV infection and human diseases remains elusive. However, severe HBoV infections have been recently reported in pediatric patients [13]–[15]. Mitui et al. reported that HBoV1 and HBoV2 DNA was the only pathogen nucleic acid detected in the cerebrospinal fluid specimens from children with severe encephalitis in Bangladesh [13], and Körner et al. confirmed HBoV infection in an 8-month-old girl with hypoxia, respiratory distress, wheezing, cough, and fever in Germany [14]. In addition, a case of life-threatening HBoV infection has been described in a pediatric patient with pneumothorax and acute respiratory failure in Slovenia [15]. These cases indicate that HBoVs may be etiological agents that can lead to severe and life-threatening diseases. In addition, a more recent longitudinal study of healthy children from infancy to early adolescence indicated that HBoV1 primary infection is significantly associated with ARTIs and otitis [16]. In light of these studies, convenient diagnostic tools for HBoV infections will be helpful for assessing the role HBoVs play in respiratory infections.

The most efficient and effective diagnostic tests should be capable of detecting multiple HBoV species in a single sample. One way to develop such a test is through the identification of an immunogenic epitope that is conserved across many HBoV species. The HBoV genome encodes four proteins–two nonstructural proteins (NS1 and NP1) and two overlapping capsid proteins (VP1 and VP2) [1]. In fact, recent evidence suggests that common immunoreactive epitopes among HBoVs may exist within the VP2 protein. For example, the VP2 protein contains the major antigen of HBoV and can form empty virus-like particles (VLPs). HBoV VLPs, which are similar in morphology and antigenicity to virions, have been successfully used as antigens for detecting antibodies against HBoVs [10], [17], [18]. Additionally, the homologies of the amino acid (aa) sequences of the HBoV1–4 VP2 are high – aa sequence identities of VP2 are about 77–78% between HBoV1 and HBoV2–4, 88–90% between HBoV2 and HBoV3–4,and 90.7% between HBoV3 and HBoV4. Further, recent studies have shown strong serological cross-reactivities among HBoV1–4 VP2 VLPs [9], [10]. These data suggest that common immunoreactive epitopes among HBoVs may exist and highlight the potential for its use as a diagnostic tool for HBoV infection. However, the epitopes in the HBoV VP2 proteins have not been finely mapped.

Gene fragment phage display libraries (GFPDLs) have become a powerful tool to identify and map antigen epitopes following natural exposure to or vaccination against pathogens, and has contributed largely to infectious disease diagnostics, vaccine designs, and antibody repertoires evaluation [19]–[21]. In this study, we constructed HBoV1–3 GFPDLs to identify the VP2 epitopes recognized by mouse and human antisera. By comparing the epitope recognition maps, we identified conserved VP2 epitopes that demonstrated the potential of HBoV peptides for undifferentiating, single-well detection of antibodies against any of the four known HBoV species.

## Results

### HBoV VP2 epitopes identified by GFPDL panning

**Figure 1:**
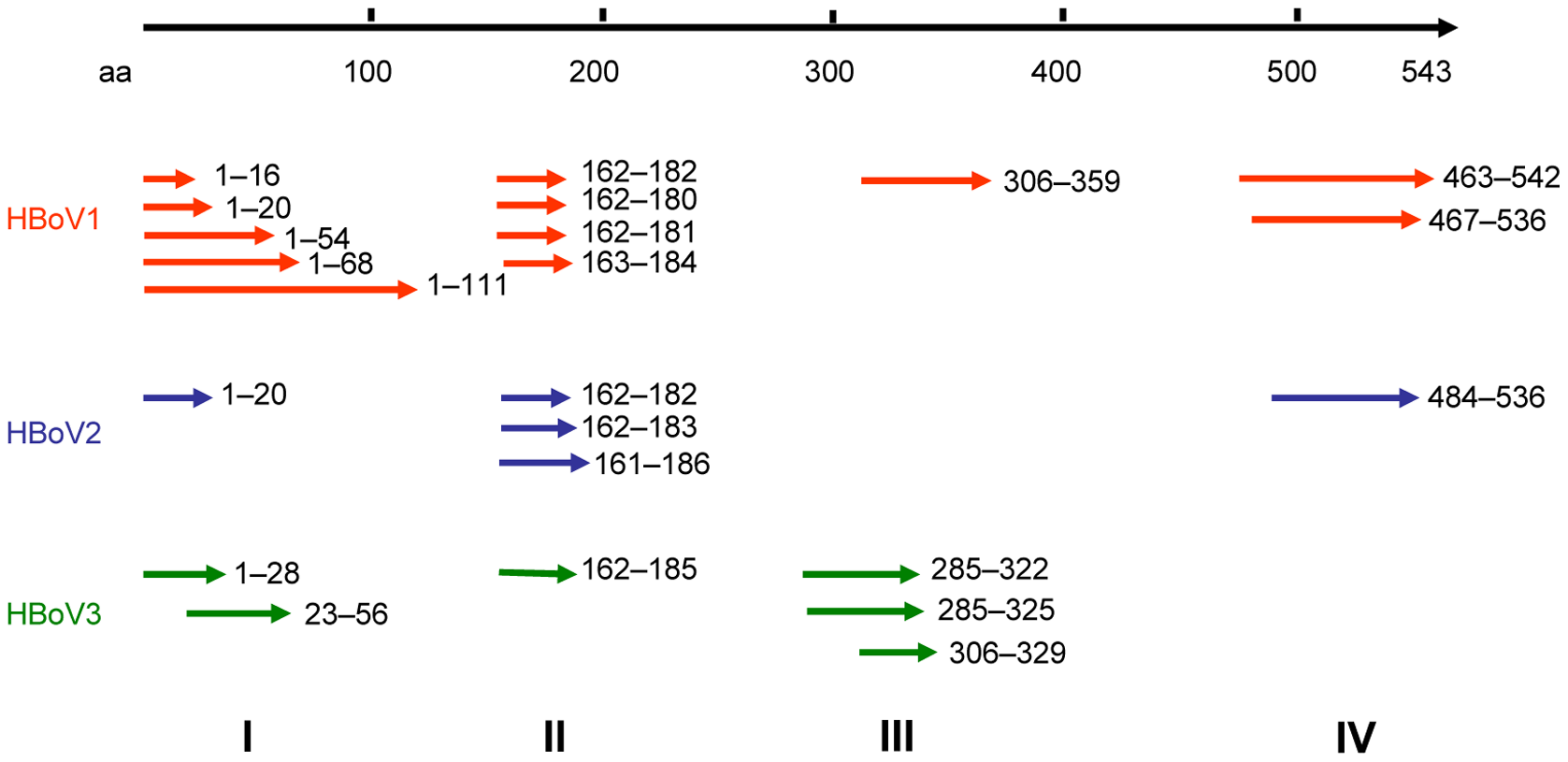
Epitopes identified in HBoV VP2 proteins by GFPDL screening. HBoV epitopes were identified by GFPDL panning using mouse sera obtained after immunization with HBoV1, 2, and 3 VP2, and human sera positive for HBoVs. Amino acid numbers correspond to HBoV strain 111-BJ07 (GenBank accession number JQ240469).

To identify antigenic clusters, we performed panning of GFPDLs with mouse antisera against VP2 protein of HBoV1, 2 and 3, as well as human sera positive for HBoVs. In the GFPDL screening, the phage clones that harbor inserts encoding epitopes, which can be recognized by mouse and human sera, were obtained in the affinity selections and were amplified by PCR for sequencing. Only the clones with a frequency of ≥2 in the panning were regarded as the positive clone [20]. Using these methods, we identified four antigenic clusters (I–IV), distributed across the VP2 protein (Figure 1). Of note, the sequences we identified share similar positions in HBoV1, 2, and 3. In addition, we found that the epitopes located at aa 1–20 (designated P1) in cluster I, and aa 162–180 (designated P2) in cluster II were present in HBoV1, 2, and 3. However, the epitopes in cluster III (P3) were only present in HBoV1 and HBoV3, and the epitopes in cluster IV (P4) were only present in HBoV1 and HBoV2. These results suggest that P1 and P2 may be conserved immunoreactive epitopes among HBoV species.

### Immunogenicity of the immunodominant HBoV peptides

**Figure 2:**
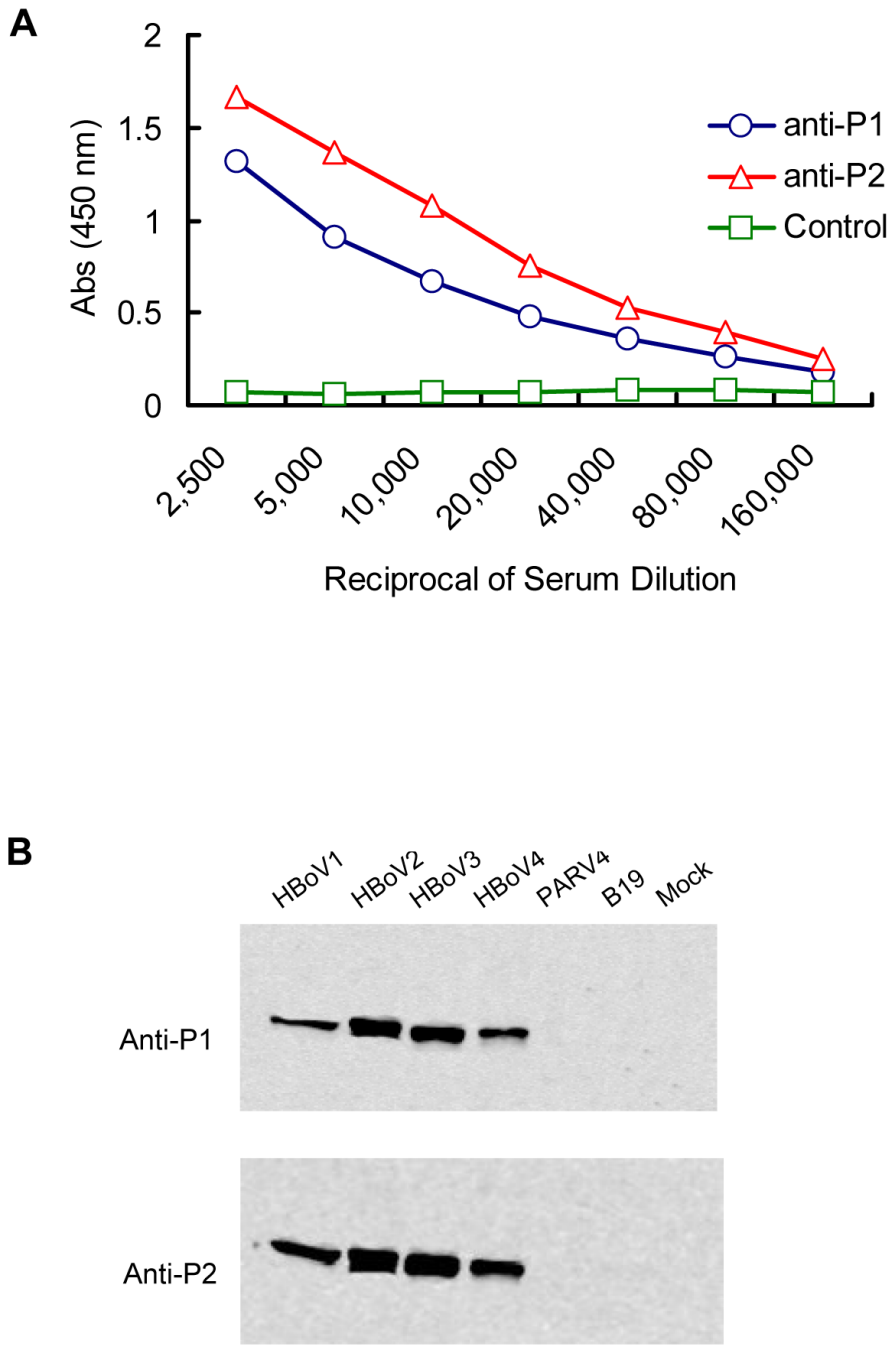
Antigenic characterization of potential HBoV epitopes P1 and P2. (A) Titers of IgG antibody against P1 and P2 in mouse sera. The titers of mice sera were determined as a series of two-fold dilutions by ELISA. (B) The immunological cross-reactivity was analyzed between mice antisera against P1 or P2 with virus-like particles (VLPs) of HBoV1, 2, 3, and 4 by Western blot. The VLPs of human parvovirus B19 and PARV4 were used as controls.

To test the immunogenicity of the VP2 segments identified by GFPDL panning, we synthesized two immunodominant peptides–P1 and P2, and used the keyhole limpet hemocyanin (KLH) conjugates of these peptides to immunize mice (Table 1). After three rounds of immunization, we effectively generated specific IgG antibodies against P1 and P2, and achieved titers as high as 1∶160,000 (Figure 2A), indicating that the P1 and P2 peptides are strongly immunogenic peptides.

**Table 1:** Sequences of the peptides conjugated with KLH carrier for mice immunization.

Designation	Position	Sequence
P1-KLH	1–20^a^	KLH-CMSDTDIQDQQPDTVDAPQNT
P2-KLH	162–180^a^	KLH-CEHAYPNASHPWDEDVMPDL

a Indicated as the position corresponding to the VP2 protein of HBoV1 strain 111-BJ07

(GenBank accession number JQ240469).

### Conservation of P1 and P2 among HBoV species

To verify whether the peptides of P1 and P2 are conserved epitopes among the known HBoV species (HBoV1–4), we subjected the purified VP2 VLPs of HBoV1–4, human parvovirus B19 (B19), and human parvovirus 4 (PARV4) to Western blot analysis using mouse sera against P1 and P2. We found that the polyclonal antibodies against the two peptides reacted with HBoV1–4 VLPs rather than with the VLPs of B19 and PARV4 (Figure 2B). These results suggest that the peptides of P1 and P2 are HBoV specific and conserved among HBoV1–4.

**Figure 3:**
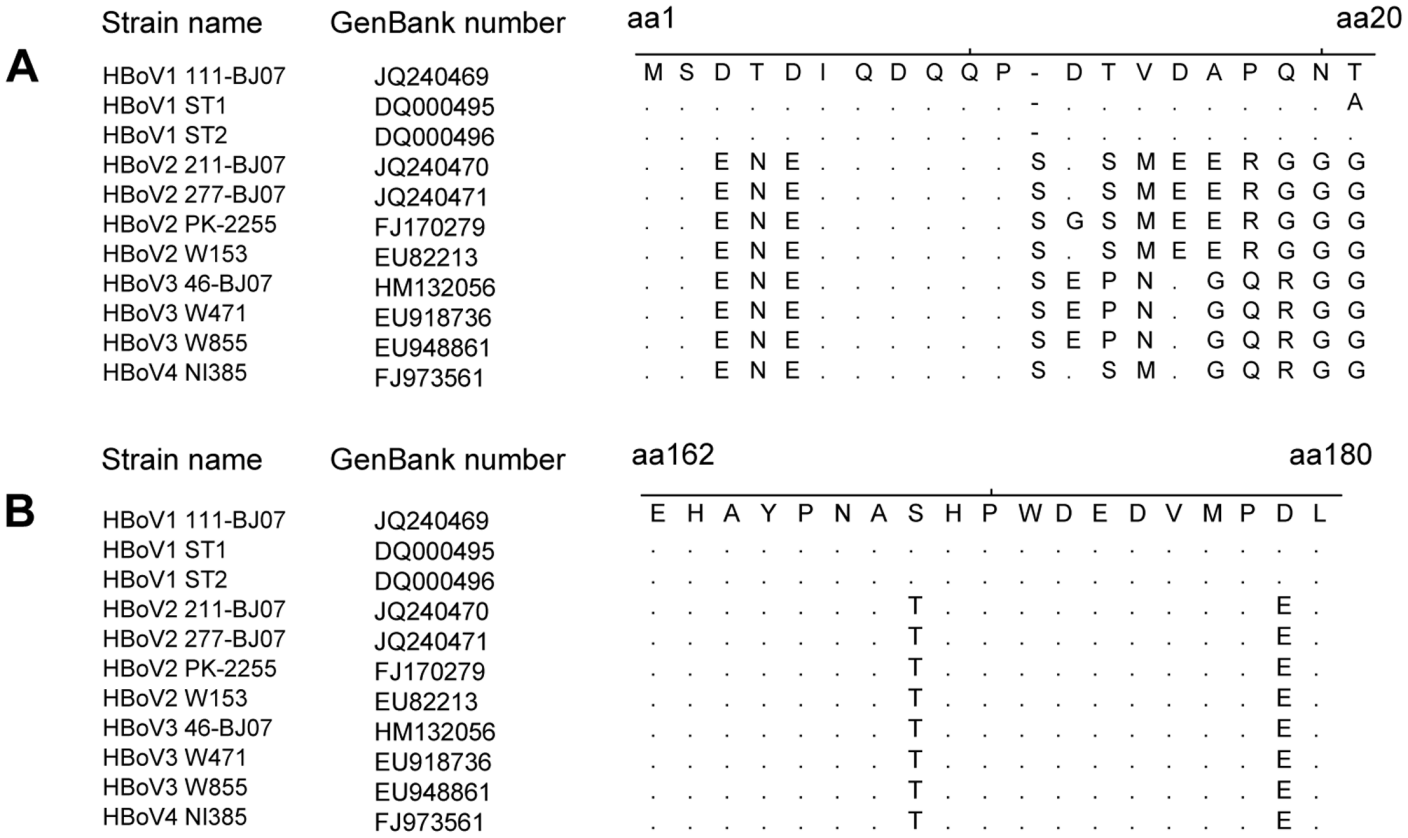
Alignment between the amino acid sequences of the peptides P1 and P2 with the corresponding representative VP2 sequences of HBoV species 1–4. Sequences of aa1 to 20, 162 to 180, according to the VP2 protein of HBoV strain 111-BJ07 (GenBank accession number JQ240469), are aligned with the corresponding region of multiple VP2 proteins of HBoV1–4 species using MEGA 4.0 software [28].

To confirm the conservation of P1 and P2 among different HBoV species further, we aligned the amino acid sequences of P1 and P2 with the corresponding amino acid sequences of HBoV1–4. We found that ^1^MS^2^ and ^6^IQDQQP^11^ in the P1 epitope have 100% amino acid sequence identities among HBoV1, 2, 3, and 4; while ^162^EHAYPNA^168^, ^170^HPWDEDVMP^178^, and L^180^ in the P2 epitope showed 100% amino acid sequence identities among HBoV1, 2, 3, and 4 (Figure.3). These findings further indicate that the aa sequence of P1 and P2 are conserved among HBoV1–4.

### Precise mapping of the HBoV epitopes

To precisely map the epitopes contained in P1 and P2, we performed a peptide-inhibition ELISA assay to examine the abilities of a panel of short peptides derived from P1 and P2 to inhibit the binding of mouse antisera to the parent peptides.

**Figure 4:**
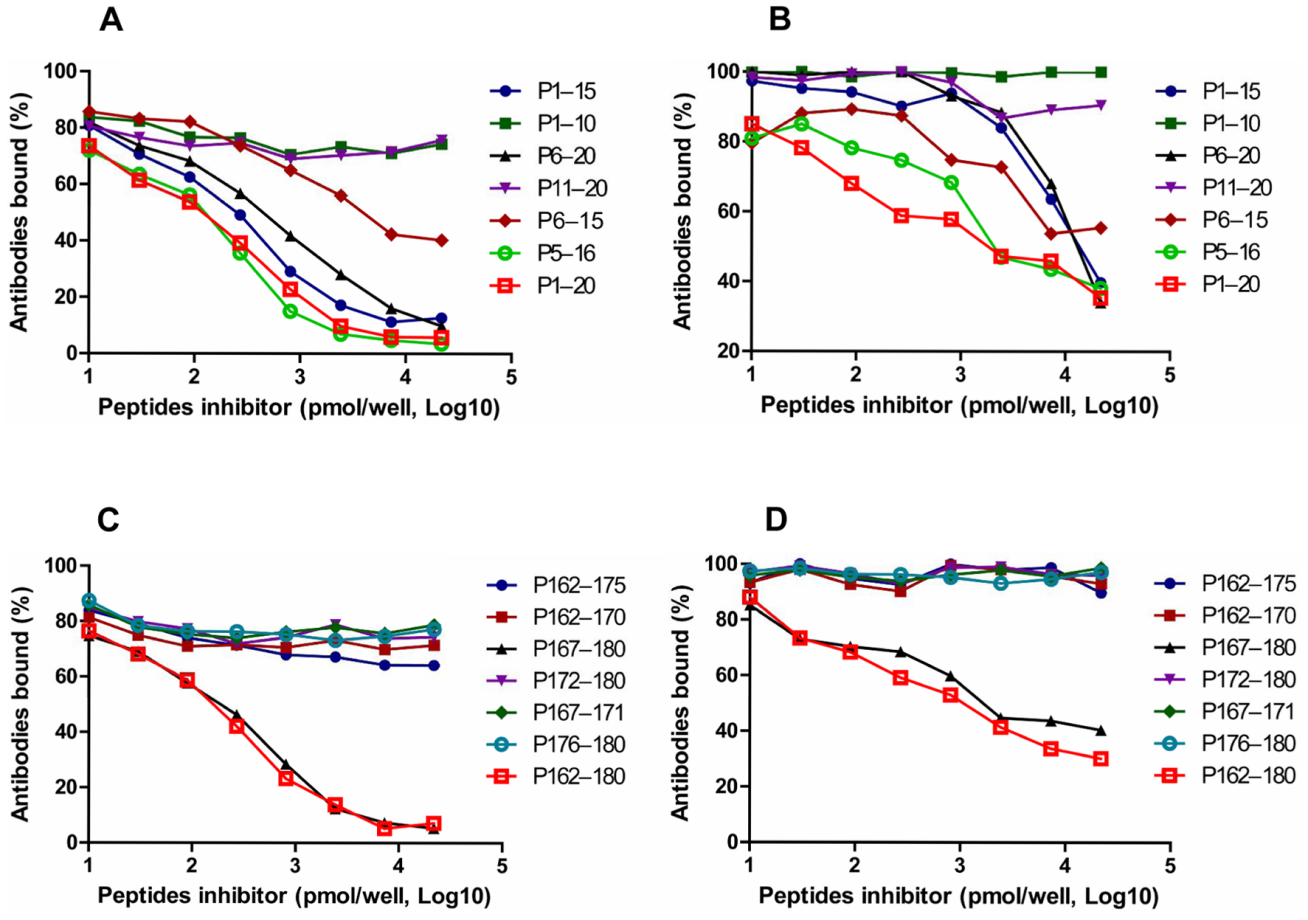
Inhibition of the binding of serum against P1 and P2 to parental peptide P1 and P2 by peptide homologs. Mouse antiserum samples induced by the P1-KLH/P2-KLH conjugate were tested by ELISA for their ability to bind to the P1/P2 peptide in the presence of dilutions of short peptide homologs to the P1 (A) and P2 (C) peptides. Human serum samples positive for HBoVs were tested by ELISA for their ability to bind to the P1/P2 peptide in the presence of dilutions of short peptide homologs to the P1 (B) and P2 (D) peptides.

We found that the binding of P1 to anti-P1 was inhibited by the shorter peptides ^1^MSDTDIQDQQPDTVD^15^ and ^6^IQDQQPDTVDAPQNT^20^ (Figure 4A). However, the peptides ^1^MSDTDIQDQQ^10^ and ^11^PDTVDAPQNT^20^ lost the ability to block the binding of anti-P1 antibody to P1, which suggests that the peptide ^6^IQDQQPDTVD^15^ is likely the critical peptide fragment for P1 antigenecity. Subsequently, we synthesized peptide ^6^IQDQQPDTVD^15^ to confirm its ability to block the binding of anti-P1 antibody to P1 by peptide-inhibition ELISA. We found that the ^6^IQDQQPDTVD^15^ peptide could inhibit the binding of anti-P1 to P1, though inhibition was less than that achieved with P1. To clarify whether the deleted N and C terminal amino acids could provide part of role in the binding to P1 antibody, we used peptide ^5^DIQDQQPDTVDA^16^ to perform a peptide-inhibition ELISA. We found that the peptide ^5^DIQDQQPDTVDA^16^ achieves inhibition that is comparable to P1 (Figure 4A), indicating that the deleted N and C terminal amino acids participate in the binding of P1 to anti-P1.

Furthermore, we observed that the binding of anti-P2 to P2 was blocked by the shorter peptide ^167^NASHPWDEDVMPD^180^ (Figure 4C). However, the peptides ^172^WDEDVMPDL^180^ and ^162^EHAYPNASHPWDED^175^ lost the ability to inhibit the binding of anti-P2 to P2, which suggests that the peptides ^167^NASHP^171^ and ^176^VMPDL^180^ are also likely a critical part of the binding site for P2 antibody. To confirm their ability to block antibody binding, the peptides ^167^NASHP^171^ and ^176^VMPDL^180^ were used in a peptide-inhibition ELISA assay. We found that the peptides of ^167^NASHP^171^ or ^176^VMPDL^180^ alone was not able to block the binding of anti-P2 antibody to P2 (Figure 4C), suggesting additional amino acids among the peptides are also involved in the binding of P2 antibody.

To verify these findings in human humoral responses, we performed P1 and P2 peptide-inhibition ELISA assays using human sera positive for HBoVs. Interestingly, we achieved similar results with human and mouse sera (Figure 4B, 4D), except that the inhibition of antibody binding was less than that achieved with mouse sera. These results suggest that the P1 and P2 peptides can also stimulate humoral responses in humans. However, epitopes recognized and presented in the process of antigen presentation may be different in humans and mice, leading to differences in antigen structures essential for antibody binding.

### Performance of peptide-IgM ELISA for HBoV antibody detection

As the P1 and P2 epitopes are antigenic and conserved among HBoV species, we developed an ELISA to detect IgM against HBoV and assessed its performance as a diagnostic tool by using clinical serum samples. Based on the findings from our epitope mapping experiments, P1 and P2 were used as coating antigens to keep the binding capacity of the epitopes. We compared the performance of P1, P2, or P1+P2 in detecting IgM (P1-IgM ELISA, P2-IgM ELISA, P1+P2-IgM ELISA), respectively. Acute-phase serum samples were obtained from 89 children with acute LRTIs from day 1 to 3 after onset of fever. The HBoV VLP IgM ELISA test was used as a positive control. Overall, results from the P1 and P2 IgM ELISAs were comparable to those achieved with the VLP IgM ELISA. Specifically, results matched 94.4% for P1-IgM ELISA, 95.5% for P2-IgM ELISA, and 95.5% for P1+P2-IgM ELISA (Table 2), indicating that the two methods have good correlation. However, the sensitivity and specificity of P1-, P2-, and P1+P2-IgM ELISA versus HBoV VLPs IgM ELISA was 72.7% and 97.4%, 72.2% and 98.7%, and 90% and 97.4%, respectively. These data suggest that the P1+P2-IgM ELISA methods have stronger potential to detect IgM antibody against HBoVs than the assays using a single peptide.

**Table 2:** Comparison of peptide ELISA and VLP ELISA in detection of HBoV IgM antibodies.

	VLP		
P1	Positive	Negative	Total
Positive	8	2	10
Negative	3	76	79
Total	11	78	89
P2			
Positive	8	1	9
Negative	3	77	80
Total	11	78	89
P1+P2			
Positive	10	3	13
Negative	1	75	76
Total	11	78	89

## Discussion

In this study, we identified immunodominant epitopes of HBoV VP2 proteins using the GFPDL assay. Our findings show that peptides P1 (aa 1–20) and P2 (aa 162–180) contain conserved epitopes among HBoV1–4, and elicit high titer antibodies in mice. They were also recognized by human polyclonal antibody. We also showed that though either P1 or P2 can be used in an ELISA to detect the IgM antibodies against HBoVs in acute phase sera from ARTI patients, the P1+P2-IgM ELISA showed higher sensitivity and specificity than the assays using single peptide alone. Thus, in addition to mapping the linear cross-reactive B cell epitopes areas among the four known HBoV species, our studies provide the basis for a potential diagnostic tool for HBoV infections.

Many patients show HBoVs persistence in the nasopharynx, which makes PCR-based diagnosis problematic [22]. Hence, immunological assays can be a useful method for diagnosing HBoV infections. Whereas peptide-based detection of antibodies involves relatively straightforward techniques, conventional VLP-based IgM and IgG ELISAs require laborious antigen preparation. As such, virus epitope-based peptide antigens have proven to be useful for laboratory diagnosis of some viral infections, including Influenza A virus [23], Dengue virus [24], and West Nile virus [25], etc. The results of the peptide ELISA tests in our study support the use of epitope-based peptides as serological reagents in the diagnosis of HBoV infection and suggest that the combination of two epitope-based peptides may increase the sensitivity of this method. In addition, we performed peptide P3- and P4- IgM ELISAs using acute-phase serum samples from children with acute LRTIs. However, we found that the sensitivity of these ELISA assays was low (45.4% for P3 and 54.5% for P4, respectively). As such, peptides P3 and P4 were not pursued as an approach for antibody detection.

In this study, we only screened the IgM antibody against HBoV from acute LRTI patients, as we would like to develop an alternative diagnostic tool for acute HBoV infections. Thus, the performance of this assay for IgG tests should be verified in future studies. Furthermore, we found that the absorption values at 450 nm of the peptide-based ELISA assay used to detect HBoV-infected serum samples were lower than that of HBoV VLP-based ELISA, as is the case of a serologic test based on Dengue virus B-cell epitopes [24]. Hence, it will be necessary to improve the detection sensitivity of the peptide ELISA method in future studies. For instance, conjugating the HBoV peptides to a carrier protein, such as bovine serum albumin (BSA), may be helpful to increase the absorbance in the ELISA assay [26].

In summary, we have identified two immunodominant epitopes that are conserved among all known HBoV species. These findings provide insight into the cross-reactivities of HBoV1–4. The study also provides a basis for developing new diagnostic tools for HBoV.

## Materials and Methods

### HBoV polyclonal antibodies and serum samples

Mouse antisera against the VP2 proteins of HBoV1, 2 and 3 were produced as previously described [10]. The human sera were identified as positive for HBoVs using VLP ELISA [10]. Acute-phase serum samples (taken within 3 days after the onset of fever) were collected from 89 children (median age 14 months; range of 1 month to 13 years) with acute lower respiratory tract infections (LRTIs) when they were hospitalized at the Beijing Children's Hospital.

Written informed consent was obtained from all guardians on behalf of children. This study was approved by the ethical review committee of the Institute of Pathogen Biology, the Chinese Academy of Medical Sciences, and by Beijing Children's Hospital.

### Panning of GFPDLs

As the HBoV1, 2 and 3 are the major HBoV species detected in humans, we screened the epitopes in VP2 by GFPDLs using the full-length VP2 genes of HBoV1, 2 and 3 (GenBank accession numbers: JQ240469, JQ240470 and HM132056, respectively) [10]. The gIIIp display-based phage vector pCom3XV (a gift from Dr. Yuxian He at MOH Key Laboratory of Systems Biology of Pathogens, IPB, CAMS) was used to express the desired polypeptide as a gIIIp fusion protein. The library affinity selection was performed as previously described using mouse antisera against VP2 proteins of HBoV1, 2 and 3, and human polyclonal sera [19]. For GFPDL panning using mouse and human antisera, equal volumes of sera collected from five mice or five humans were pooled.

### Peptides and conjugates

The immunodominant peptides –P1 (^1^MSDTDIQDQQPDTVDAPQNT^20^), and P2 (^162^EHAYPNASHPWDEDVMPDL^180^), corresponding to the VP2 protein of HBoV1 strain 111-BJ07 (GenBank accession number JQ240469), were selected for immunization experiments (Table 1). To improve their immunogenicity, these peptides were synthesized and conjugated to KLH (Sigma, St. Louis, MO). To fine tune the epitope map of antibody binding sites on P1 and P2 peptides, a series of short peptides (unconjugated) homologous to the P1 and P2 peptides were synthesized by Sangon Biotech (Shanghai, China). Each peptide was purified to achieve a purity of ≥95% by high performance liquid chromatography. Each peptide was then verified by mass spectrometry.

### Animal immunizations

Female BALB/c mice, six to eight weeks old, were subcutaneously immunized with 100 µg peptide-KLH conjugates in Freund's complete adjuvant (Sigma). The mice were boosted twice at 2-week intervals with 50 µg peptide-KLH conjugates in Freund's incomplete adjuvant (Sigma). Serum samples were collected two weeks after the last immunization. This study was carried out in strict accordance with Chinese government's animal experiment regulations. All animal experiments were performed in the facilities of the Institute of Laboratory Animal Sciences, Chinese Academy of Medical Sciences (ILAS, CAMS), and all experimental procedures were approved (license number SCXKJ2012-0001) and supervised by the Animal Protection and Usage Committee of ILAS, CAMS.

### ELISA

Indirect ELISA was used to detect mouse antibodies against P1 and P2, as described elsewhere [23]. Briefly, ninety six-well microtiter plates (Corning Costar, Acton, MA) were coated with peptides P1 and P2 at 1 µg/well in 0.1 M carbonate buffer (pH 9.6) at 4°C overnight.

Peptide-inhibition ELISA assays were performed to evaluate the reactivity of the P1- and P2-derived short peptides with the corresponding antibody against P1 and P2, as described elsewhere [23].

The reactivities of the synthetic P1 and P2 peptides (1 µg/well) with acute-phase serum samples from acute LRTI patients were also determined by ELISA. ELISA performed using coating antigens of HBoV1–4 VLPs (VLP ELISA) were used as positive controls, where 50 ng/well of each HBoV species VLPs were used to coat 96-well microtiter plates (Corning Costar) in 0.1 M carbonate buffer (pH 9.6) at 4°C overnight. The plates coated with peptides or VLPs were then blocked with 300 µL 1% (w/v) bovine serum albumin (BSA, Sigma) in PBS at 37°C for 2 h. Acute-phase serum samples (100 µL) were tested at 1∶100 dilutions with HBoV peptides or HBoV1–4 VLPs, simultaneously. After washing five times with PBST (300 µL; PBS containing 0.5% Tween-20), HRP-conjugated goat anti-human IgM (100 µL; Sigma) was added to the plates at a dilution of 1∶40,000 and the plates were incubated at 37°C for 1 h. Plates were washed five times with PBST (300 µL) and developed with substrate solutions A and B (100 µL; Wantai Biotech, Beijing, China). The absorbance of each serum sample was read at 450 nm using a multifunctional microplate reader, SpectraMax M5 (Molecular Devices, Sunnyvale, CA). As the VLP ELISA is designed to test for all four HBoV species in one well, a positive result indicates that the sample is infected with at least one of the four HBoVs (HBoV1, HBoV2, HBoV3 or HBoV4) and a negative result indicates that the serum sample is negative for all four HBoVs.

To determine the cut-off value, all the absorbance values below a provisional cut-off of 0.20 were taken and their mean and standard deviations (SD) were calculated as previously reported by Kahn and Hustedt [18], [27]. All samples with values above 0.18 and 0.15 (mean + 3 SD) were considered positive for HBoV VLPs IgM ELISA and peptide IgM ELISA.

### Western blot analysis

HBoV1–4 VLPs, B19 VLPs, and PARV4) VLPs were expressed and purified, as described previously [10]. The VLPs of HBoV1–4, B19, and PARV4 were loaded on a 12% SDS-PAGE gel. The gels were transferred to a nitrocellulose membrane (Pall, Port Washington, NY) and blocked with 5% nonfat dry milk. The P1 and P2 peptide antibodies produced in mice were applied followed by incubation with the corresponding goat anti-mouse IRDye Fluor 800-labeled IgG secondary antibody (1∶10,000) (Li-Cor, Lincoln, NE). The membranes were scanned by the Odyssey Infrared Imaging System (Li-Cor) and analyzed with Odyssey software.
